# FGF21 Response to Sucrose is Associated with BMI and Dorsal Striatal Signaling in Humans

**DOI:** 10.1002/oby.23432

**Published:** 2022-05-02

**Authors:** Jasmin M. Alves, Alexandra G. Yunker, Shan Luo, Kay Jann, Brendan Angelo, Alexis DeFendis, Trevor A. Pickering, Alexandro Smith, John R. Monterosso, Kathleen A. Page

**Affiliations:** 1 Division of Endocrinology, Department of Medicine, Keck School of Medicine, University of Southern California, Los Angeles, CA 90089.; 2 Diabetes and Obesity Research Institute, Keck School of Medicine, University of Southern California, Los Angeles CA 90089.; 3 Department of Psychology, University of Southern California, Los Angeles, CA 90089, USA; 4 Mark & Mary Stevens Neuroimaging & Informatics Institute, Keck School of Medicine, University of Southern California, Los Angeles, CA 90089; 5 Department of Preventive Medicine, Keck School of Medicine, University of Southern California, Los Angeles, CA 90089

**Keywords:** Fibroblast growth factor 21 (FGF21), sugar, obesity, brain, sucrose

## Abstract

**Objective:**

This study examined associations between BMI and dietary sugar intake with sucrose-induced fibroblast growth factor 21 (FGF21) and whether circulating FGF21 is associated with brain signaling following sucrose ingestion in humans.

**Methods:**

68 adults (29 male; 23.2 ± 3.8 years; BMI 27.1 ± 4.9 kg/m^2^) attended visits after a 12h fast. Plasma FGF21 was measured at baseline, +15, +30, and +120min after sucrose (75g in 300 mL water). Brain CBF responses to sucrose were measured using arterial spin labeling magnetic resonance imaging (MRI).

**Results:**

Higher circulating FGF21 levels were associated with reduced blood flow in the striatum in response to sucrose (ß=−7.63, p=0.03). This association was greatest among persons with healthy-weight (ß=−15.70 p=0.007) and attenuated in people with overweight (ß=−4.00, p=0.63) and obesity (ß=−12.45, p=0.13). BMI was positively associated with FGF21 levels in response to sucrose (ß=0.53, p=0.02). High vs low dietary sugar intake was associated with greater FGF21 responses to acute sucrose ingestion in healthy-weight individuals (ß=8.51, p=0.04), but not individuals with overweight or obesity (p>0.05).

**Conclusions:**

Our correlative findings support evidence in animals showing FGF21 acts on the brain to regulate sugar consumption through a negative feedback loop.

## Introduction

Obesity rates have risen dramatically over the last three decades, posing a significant challenge to public health (1). A growing body of work has linked excessive sugar consumption to increased risk of obesity and metabolic disorders, which highlights the importance of understanding the biological mechanisms that regulate sugar consumption (2). Emerging evidence indicates that the hormone fibroblast growth factor 21 (FGF21) plays an important role in the regulation of sugar appetite and sweet taste preference (3). In humans, FGF21 has been shown to robustly increase after the ingestion of fructose (4), but its rise is attenuated in response to glucose (5), likely due to differences in hepatic metabolism between the two monosaccharides. Notably, fructose-stimulated FGF21 levels were shown to be increased in adults with metabolic syndrome compared to healthy participants (4), and plasma FGF21 levels following glucose administration were found to be higher in people with obesity compared to healthy-weight (6), suggesting that metabolic impairments affect carbohydrate-induced FGF21 secretion. Fructose is rarely consumed in isolation; whereas, sucrose, which is a disaccharide composed of equal parts glucose and fructose and commonly known as “table sugar”, is frequently added to foods and beverages. In addition to the previous findings showing fructose-linked increases in FGF-21 secretion, Søberg and colleagues recently demonstrated that plasma FGF21 also increased markedly after an oral sucrose load, but this study was limited to adults with healthy weight so the association between BMI and FGF21 response to sucrose remains unknown (7).

Additional studies have shown that FGF21 gene variants are associated with a preferential increase in dietary carbohydrate intake (8), particularly sweet-containing foods (7,9,10). Moreover, FGF21 was found to selectively reduce appetite for simple sugars without changing intake of other macronutrients or overall calorie intake (3). Mechanistic studies in rodents and non-human primates demonstrated that FGF21 reduces sweet taste preference through its effects on striatal dopamine signaling (11) and hypothalamic neurons (3,12). Collectively, these findings are suggestive of FGF21 acting as a down-regulator of subsequent sweet consumption through its effects on the central nervous system and suggest a role of FGF21 in nutrient-specific appetite regulation.

Given the recent evidence indicating that FGF21 plays a role in the regulation of sugar intake, which is likely mediated through a central nervous system mechanism (3,11,12), and prior evidence suggesting that FGF21 is affected by obesity and diet (13,14), we aimed to test the hypotheses that greater circulating FGF21 levels would be negatively associated with brain responses to oral sucrose in regions involved in appetite regulation, and that BMI and dietary added sugar intake would be independently associated with sucrose-induced increases in FGF21 in humans.

## Materials and Methods

### Study Overview

The data reported here are part of the larger Brain Response to Sugar II study examining neuroendocrine responses to high-reward foods (NCT02945475). We used existing data from this trial in the current analysis (trial protocol and flow diagram can be found in online digital repository (15)). Recruitment occurred between July 2016 and March 2020. The sample size for the larger trial was determined based on our prior work that provided a within subject effect of 0.60 SD for differences in brain cerebral blood flow responses to oral fructose vs glucose (16). The Brain Response to Sugar II within-participant randomized crossover trial included four drink conditions (see digital repository for full trial protocol (15)), but the data analyzed for this manuscript included only the sucrose drink condition. As part of the larger Brain Response to Sugar II study, participants were recruited into three BMI groups (healthy weight (HW): 18.5–24.9 kg/m^2^, overweight (OW): 25–29.9 kg/m^2^, and obesity (OB): ≥ 30 kg/m^2^) based on Centers for Disease Control and Prevention (CDC) criteria (17). (15). Participants provided written informed consent compliant with the University of Southern California Institutional Review Board (IRB #HS-09–00395).

All visits were conducted in the morning after a 12h overnight fast. During the screening visit, we assessed eligibility for participation in the study and demographic information and anthropometric measurements were obtained. Participants were right-handed, nonsmokers, weight-stable for at least 3 months prior to the study visits, non-dieters, not on any medication (except oral contraceptives), and with no history of diabetes or other medical diagnoses, eating disorders, illicit drug use, or habitual alcohol use. Height was measured to the nearest 0.1cm using a stadiometer and weight to the nearest 0.1kg using a bioelectrical impedance analysis scale (Model no. SC-331S, TANITA Corporation of American, Inc.), and BMI was calculated as weight in kilograms divided by height in meters squared.

Participants arrived at the Dornsife Cognitive Neuroimaging Center of University of Southern California at 8:00AM for the MRI visit. During this visit, participants consumed a standard 75g sucrose load, mixed with 0.45g of non-sweetened zero calorie cherry flavoring (Kraft Foods Kool-Aid^®^ Unsweetened Cherry Drink Mix) for palatability, dissolved in 300mL of water. Blood samples were collected at baseline (0min), 10min, 35min, and 120min post-sucrose and plasma FGF21, glucose, and insulin were measured. MRI sequences included a T1 structural scan (for anatomical registration) and an arterial spin labelling (ASL) scan, followed by participants exiting the scanner to consume the sucrose drink within two min (in order to reduce variability in timing of drink effects). After consuming the sucrose drink, participants re-entered the scanner and underwent two additional ASL sequences at approximately 5 and 30min post-drink ingestion. A food cue task measuring blood oxygen level dependent (BOLD) signaling was also collected as a part of the larger study, but not included in the current analysis. Female subjects underwent study visits during the follicular phase of their menstrual cycles.

### Metabolite and Hormone Analysis

Plasma FGF21 (pg/ml) was measured using a human FGF21 ELISA kit (Millipore, Billerica, MA). Plasma glucose and insulin were included as co-variates in the statistical analyses because they have both been shown to affect brain signaling (16,18). Plasma glucose (mg/dl) was measured enzymatically using glucose oxidase (YSI 2300 STAT PLUS Enzymatic Electrode-YSI analyzer, Yellow Springs Instruments) and plasma insulin (pg/ml) was measured via Luminex multiplex technology (Millipore, Billerica, MA). In order to represent overall response to sucrose ingestion, we calculated 120min total area under the curve (AUC) for plasma FGF21 using the trapezoid method, which is calculated by adding the areas under the graph between each pair of consecutive observations, as previously described (19).

### Habitual Added Sugar Intake Assessment

Diet was assessed using the multi-pass 24-hour dietary recall, which is a validated method that provides detailed information on food and beverages consumed over the previous 24-hour period (20). Each dietary interview was administered by a trained staff member, wherein volunteers were asked to recall all food and drinks items (including meals and snacks) that they ingested during the previous 24-hours. To account for potential daily variations in dietary intake, 24-hour recalls were captured on both weekdays and weekend days. After the dietary recalls were obtained, the data was analyzed using the Nutritional Data System for Research (NDSR) software v.2018, developed by the Nutrition Coordinating Center, University of Minnesota, Minneapolis, MN, USA (21). Dietary recalls were also assessed for plausibility and quality using the method described by Jones et al. (22), and using this method, 359 dietary recalls were included in the analysis (an average of 5 dietary recalls over the course of approximately 2 months, per participant) and 5 recalls were excluded. The timing of the dietary assessments was within a 2-month period proximal to the MRI and blood sampling study visit during which FGF21 response to sucrose and brain perfusion were measured. The output from the software provided intake of overall calories and the breakdown of macronutrients, including sugar. For the purpose of this study, we analyzed the amount of added sugar in the diet as a percent of total calories, and we classified participants as either high or low added sugar consumers based on the World Health Organization’s dietary recommendations for the percentage of total calories coming from added sugar: ≥10% added sugar was considered a high amount of added sugar and <10% was considered a low amount of added sugar (23).

### MRI Imaging Parameters and Analysis

Neuroimaging data were collected using a 3T Siemens MAGNETOM Prismafit MRI System, with a 32-channel head coil. After initial localizers, a baseline pulsed arterial spin labeling (pASL) scan was acquired followed by a high-resolution 3D magnetization prepared rapid gradient echo (MPRAGE) sequence (TR=1950ms; TE=2.26ms; bandwidth=200Hz/pixel; flip angle=9°; slice thickness=1mm; FOV=224mm×256mm; matrix=224×256), which was used to acquire structural images for multi-subject registration. Two additional ASL sequences were acquired at +5 min and +30 min after sucrose ingestion. The ASL sequences used the QUIPSS-II method (24). A proximal inversion with a control for off-resonance effects (PICORE) mode was employed to provide high labeling efficiency (25). The ASL sequences were acquired along with one M0 image with following parameters: field of view (FOV) = 192 mm, matrix = 64 × 64, bandwidth = 2232 Hz/Pixel, slice thickness = 5 mm, interslice spacing = 0 mm, Repetition time (TR) = 4000 ms, echo time (TE) = 30 ms, flip angle = 90°, TI1/TIs/TI2 = 700/1,800/1,800 ms, label duration = 1675 ms, slab thickness = 100mm, in-plane resolution = 3 × 3 mm^2^. The timing of the inversion pulses (TI) were optimized to reduce intravascular signal intensity at 3 T (26). The duration of the ASL acquisitions were 8 min and 18 seconds.

Pulsed ASL data was collected using magnetically tagged arterial blood. Motion was corrected (27) and slice timing correction used 53ms for CBF travel time. Tagged and untagged images were subtracted to obtain perfusion-weighted images. Bayesian Inference for ASL (BASIL) toolbox (https://fsl.fmrib.ox.ac.uk/fsl/fslwiki/BASIL), was used to analyze the perfusion data. *A priori* brain regions-of-interest (ROI) included areas implicated in sweet taste processing and/or were selected based on evidence demonstrating FGF21 neural expression: dorsal striatum, hippocampus, hypothalamus, insula, and nucleus accumbens (3,11,12,28–32). Mean CBF across the whole brain and specifically in each ROI were extracted. All ROIs were bilateral and anatomically defined using the Harvard-Oxford cortical and subcortical structural atlas found in FSL using a voxel probability threshold above 50%, except the hypothalamus which is not included in the atlas and was defined bilaterally as a 2-mm spherical ROI surrounding peak glucose-responsive voxels (33). ASL data were motion corrected and then tagged and untagged images were subtracted to obtain perfusion-weighted images (27). ASL volumes were registered to the individual participant’s T_**1**_-weighted high-resolution anatomical volume using an affine registration with 12 degrees of freedom. Atlas ROIs where then inverse-transformed to individual native space and regional CBF was extracted for each ROI and participant.

### Statistical Analysis

Descriptive statistics were completed and can be found in Table 1. Linear mixed effects regression was used to determine if the FGF21 response to sucrose varied across the timepoints, using time as a repeated measure. One-way ANOVA was used to assess differences in baseline FGF21 levels, peak sucrose stimulated FGF21 levels (120 minutes) and FGF21 (AUC)_120_ by BMI groups and habitual added sugar intake. Additionally, linear regression was used to analyze the following primary outcomes of interest: (1) associations between FGF21 and brain CBF responses to acute sucrose ingestion, and whether this relationship differed by BMI groups; (2) associations between BMI and FGF21 response to sucrose; (3) as an exploratory analysis, we examined differences in FGF21 (AUC)_120_ stratified by BMI groups and habitual added sugar intake. FGF21 response to sucrose was calculated as the total area under the curve (AUC)_120_ over the 120min testing period. We calculated total area under the curve (AUC)_35_ for plasma FGF21 and (AUC)_35_ CBF in each ROI to quantify the FGF21 and neural responses to sucrose ingestion when both neuroimaging and blood sampling were simultaneously performed. Fasting FGF21, peak FGF21 at 120min and FGF21 (AUC)_120_ were all not normally distributed, therefore a cubic-root transformation was applied to normalize their respective distributions. Age (34), sex (35), BMI and/or added sugar intake were used as covariates. For imaging analyses, mean global CBF, glucose (AUC)_35_ and insulin (AUC)_35_, were additionally used as covariates. Mean global CBF was included as a covariate to examine specificity of circulating FGF21 levels on regional CBF within brain ROIs. Insulin (AUC)_35_, and glucose (AUC)_35_ were used as covariates to account for potential effects of circulating insulin and/or glucose on neural signaling (16,18). P-values <0.05 were interpreted as statistically significant. SAS OnDemand for Academics (SAS Institute, Cary, NC USA) was used for all data analyses, with mixed effects models performed using PROC MIXED.

## Results

Sixty-eight adults (29 male, 39 female) aged 18–35 years with a BMI range between 19.2–40.3 kg/m^2^ completed blood sampling and brain MRI before and after sucrose ingestion. Forty-one were low-added-sugar (<10%) and 27 high-added-sugar (≥ 10%) consumers (Table S1). Participant characteristics are provided in Table 1.

### FGF21 response to sucrose ingestion and associations with BMI and added sugar intake

**Figure 1.**
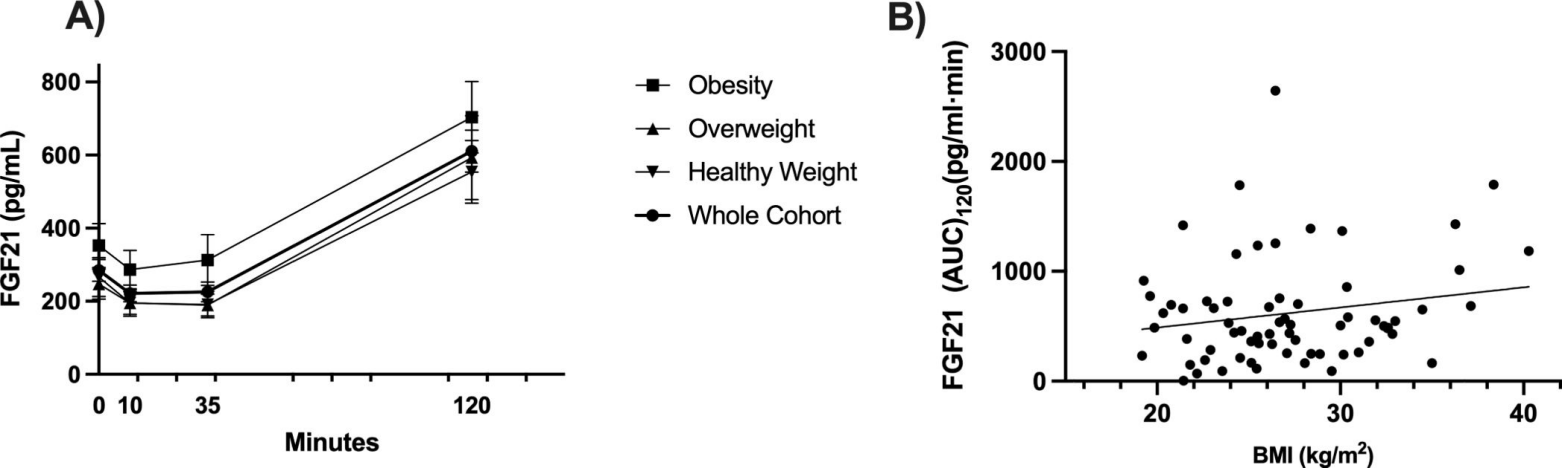
Sucrose-induced plasma FGF21 responses and associations with BMI Plasma FGF21 levels following acute sucrose consumption (N=68) at 0, 10, 35, and 120 minutes, among the cohort as a whole (circles) and stratified by body mass index (BMI) groups. Data are expressed as raw/unadjusted mean ± SEM for visual purposes, but all statistical analyses were based on cubic root transformed FGF21 values and adjusted for covariates (A). BMI was positively associated with FGF21 (AUC)_120_ response to sucrose ingestion (β=0.50, p=0.02) (B).

Among the whole cohort, mean (SD) baseline plasma FGF21 levels and FGF21 (AUC)_120_ levels are found in Table 1. Trajectories for plasma FGF21 levels among the whole cohort are shown in Figure 1A. There was a main effect of time on plasma FGF21 levels following sucrose ingestion (*F*(3,192)=84.82, p<0.0001), and post-hoc pairwise comparisons showed that peak FGF21 levels at 120 minutes post-sucrose ingestion differed from baseline FGF21 levels (*t*(192)=−11.44, p<0.0001).

Trajectories for plasma FGF21 in response to sucrose ingestion are shown by BMI groups (Figure 1A). While baseline FGF21 levels (p=0.20), peak FGF21 levels (p=0.29), and FGF21 (AUC)_120_ levels (p=0.16) did not significantly differ by BMI groups (Table S1), BMI was positively associated with FGF21 (AUC)_120_ response to sucrose ingestion (unadjusted: R=0.50, β=0.50, 95% CI: 0.06 to 0.89, p=0.02; adjusted for sex, age, added sugar intake: β=0.51, 95% CI: 0.08 to 0.94, p=0.02) (Figure 1B). BMI was also associated with baseline FGF21 levels before and after adjusting for covariates (unadjusted: β=0.10, 95% CI: 0.02 to 0.19, p=0.02; β=0.11, 95% CI: 0.02 to 0.20, p=0.02). Peak FGF21 levels were not significantly associated with BMI, but the beta was in the expected direction (unadjusted: β=0.10, 95% CI: −0.01 to 0.20, p=0.07; β=0.10, 95% CI: −0.01 to 0.21, p=0.07).

There was no significant difference between low and high added sugar consumers in baseline FGF21 levels (p=0.18), peak sucrose-induced FGF21 levels (p=0.09), or sucrose-induced FGF21 (AUC)_120_ levels (p=0.12) (Table S1). Percent calories from added sugar was also not correlated with baseline FGF21 levels (p=0.27), peak sucrose-induced FGF21 levels (p=0.25), or sucrose-induced FGF21 (AUC)_120_ levels (p=0.20) (Table S2). In exploratory analyses stratified by BMI groups, after adjusting for age and sex, we observed that among individuals with healthy weight, high vs low added sugar intake was associated with a greater FGF21 (AUC)_120_ response to oral sucrose (healthy weight, low sugar added consumers LSmean (SE): 28.15 (2.48); healthy weight, high sugar consumers LSmean (SE): 36.66 (2.96), p=0.04) (Figure S2, Table S3). Among individuals with overweight and obesity, there was no difference in FGF21 (AUC)_120_ response to oral sucrose in high vs low added sugar consumers (p>0.26) (Figure S2, Table S3). In analyses stratified by BMI group, after adjusting for age and sex, among individuals with a healthy-weight, percent calories from added sugar was significantly correlated with peak sucrose-induced FGF21 levels (Spearman R_s_=0.43, p=0.04) and sucrose-induced FGF21 (AUC)_120_ levels (R_s_=0.46, p=0.03) but not baseline FGF21 levels (p=0.13) (Table S4). There were no significant correlations in individuals with overweight or obesity (p>0.30) (Table S4).

### Negative associations between FGF21 and neural response to sucrose ingestion

**Figure 2.**
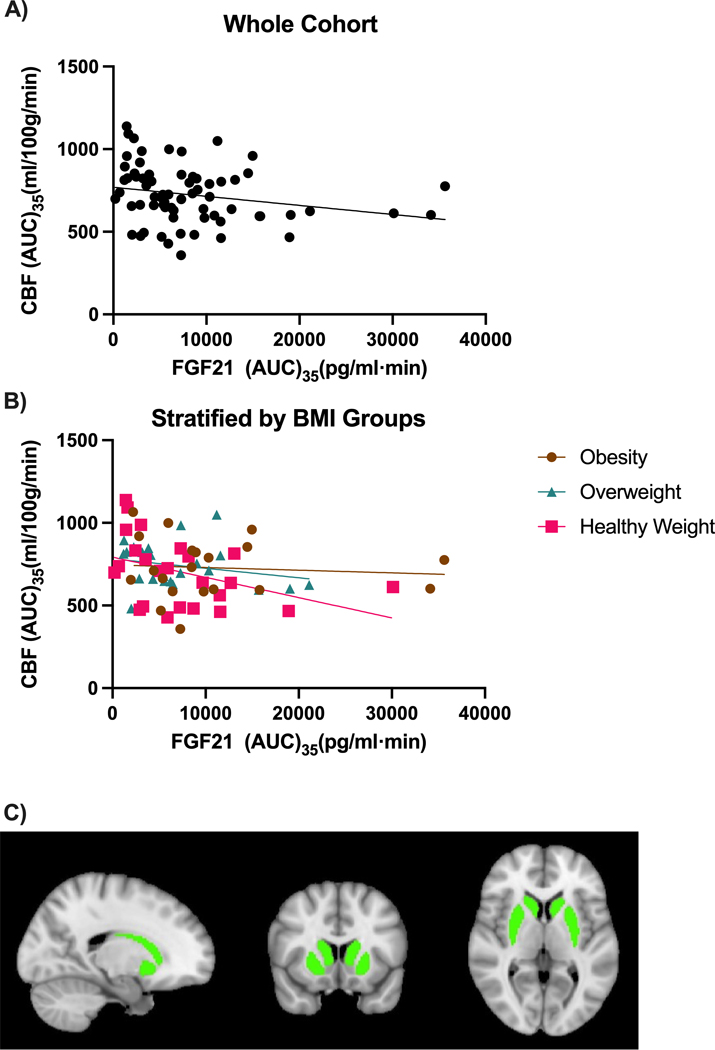
Associations between peripheral FGF21 levels and dorsal striatal cerebral blood flow Correlational graphs show the relative change in plasma FGF21 after sucrose ingestion was associated with reductions in the dorsal striatal cerebral blood flow (CBF) in response to sucrose ingestion among the cohort as a whole (A). Results stratified by BMI groups are shown in (B), where the negative relationship between plasma FGF 21 and dorsal striatal CBF was observed in healthy weight (pink), but not with overweight (green) or obesity (brown). Data are expressed as raw/unadjusted AUC_35_ values for visual purposes, but all statistical analyses were based on cubic root transformed FGF21 values and adjusted for covariates. C) Region of interest (ROI) mask of dorsal striatum [Total voxels = 2392; center voxel Left = −26, 2, 2; center voxel Right = 28 2, 2] derived from the Harvard-Oxford subcortical atlas.

Among the whole cohort, we found a negative association between FGF21 (AUC)_35_ and dorsal striatum CBF (AUC)_35_ in response to sucrose ingestion before (β=−9.12, 95% CI: −16.46 to −1.78, p=0.02) and after adjusting for age, sex, BMI groups, added sugar group, mCBF, insulin (AUC)_35_, and glucose (AUC)_35_ (β=−7.52, 95% CI: −14.38 to −0.65, p=0.04) (Figure 2A, Table S5). Similar trends were observed in the hippocampus, insula, and nucleus accumbens (Table S5). There was a significant BMI group X FGF21 (AUC)_35_ interaction on the hippocampal CBF response to sucrose (p=0.03); among individuals with healthy-weight, there was a negative association between FGF 21 (AUC)_35_ and hippocampal CBF (AUC)_35_ response to sucrose (unadjusted: β=−22.94, 95% CI: −38.94 to −6.93, p=0.01; adjusted: β=−25.21, 95% CI: −41.08 to −9.33, p=0.007), but not among individuals with overweight (unadjusted: β=−12.2, 95% CI: −29.2 to 4.8, p=0.17 adjusted =−18.13, 95% CI: −37.62 to 1.36, p=0.09) or obesity (unadjusted: β=14.35, 95% CI: −4.73 to 33.42, p=0.16; adjusted: β=−1.22, 95% CI: −15.62 to 18.06, p=0.89). We performed exploratory stratified analyses in the other brain ROIs because of extensive neuroimaging work showing that obesity affects neural responses to satiety signals (36,37). Similar to the patterns observed in the hippocampus, we also found significant negative associations between FGF21 (AUC)_35_ and CBF (AUC)_35_ after oral sucrose in the dorsal striatum (Figure 2B) and insula among individuals with healthy weight in both unadjusted and adjusted analyses, but not among individuals with overweight or obesity (Table S6).

## Discussion

To the best of our knowledge, this is the first study to investigate associations between circulating FGF21 and neural signaling following sucrose ingestion in humans. Prior studies have demonstrated that, among healthy adults, a post-prandial state leads to a decreased CBF response in the brain areas involved in food intake regulation, including the hypothalamus, insula, hippocampus, and striatum (16,36), which may be indicative of neural satiety signaling. Furthermore, altered post-prandial CBF signaling in these neural regions is associated with obesity (36–38). Previous investigators have posited that circulating FGF21 may regulate sugar consumption through a peripheral-to-brain negative-feedback loop (3,7). Our results are in line with their prediction, showing that higher circulating FGF21 resulted in larger sucrose-induced reductions in blood flow in the dorsal striatum, a brain region that plays a critical role in motivational desire for food (39). When we stratified the results by BMI group, we found that this association was strongest among participants with healthy weight compared to those with overweight and obesity. Similarly, the negative association between circulating FGF21 and hippocampal and insular responses to sucrose ingestion was also stronger in healthy-weight individuals than in those with overweight or obesity. The hippocampus acts to integrate post-nutritive signals in order to suppress appetite, and the insula has a pivotal role in gustatory processing (40,41). Prior studies have shown that excess weight gain and overweight/obesity are associated with impairments in striatal, insula, and hippocampal signaling (22,42,43). Thus, the present findings suggest that individuals with overweight and obesity might have altered FGF21-linked neural signaling in these brain regions implicated in food intake regulation, but future work is needed to test causal mechanisms.

While prior studies have shown that circulating FGF21 rises in response to acute ingestion of carbohydrates (4,5), our study is the first to examine sucrose induced FGF21 response among individuals of varying weight categories and among high vs. low added sugar consumers. Consistent with prior reports showing that circulating FGF21 levels are influenced by BMI (44–46), and that individuals with obesity and metabolic syndrome have greater FGF21 responses to ingested monosaccharides (4,6), we found that BMI was associated with greater FGF21 responses to the acute ingestion of the commonly consumed sugar, sucrose. Our data also suggest that high (vs. low) added sugar consumption is associated with a robust FGF21 response to acute sucrose ingestion among individuals with healthy weight (Figure S1). These data are in line with the concept that the body responds to chronic high sugar diets in a compensatory fashion by markedly increasing FGF21 in response to an acute sugar load, and FGF21 in turn acts as negative endocrine regulator of sugar consumption (3,7,11). While our correlative findings need to be followed up with experimental studies to determine causal mechanisms, the notion that high added sugar intake increases FGF21 production as a protective endocrine mechanism is supported by data in mice showing that a high sucrose diet increased hepatic FGF21 production and FGF21 sensitivity in brown adipose tissue, which attenuated weight gain (47). Correspondingly, human studies showed that when compared to a control diet, short-term high carbohydrate feeding led to a striking 8-fold increase in plasma FGF21 levels, which affected glucose and lipid homeostasis in healthy male volunteers (13).

It is worth mentioning, alcohol intake has also been shown to increase circulating FGF21 levels in both mice and humans (48,49), and FGF21 administration markedly reduces alcohol preference and intake in mice (48). In our study, we assessed for habitual alcohol use at our screening visit and excluded participants who reported habitual alcohol use. Furthermore, the 24-hour dietary recall assessments demonstrated that our participants consumed negligible amounts of alcohol on the day prior to the study visit, which was performed after a 12-hour overnight fast. Thus, it is unlikely that alcohol intake contributed to the circulating FGF21 levels in our study participants.

It is important to note that this was a preliminary study and the first to investigate associations between circulating FGF21 and neural signaling in humans. While our findings should be interpreted cautiously, they set the stage for future research that could examine the effects of circulating FGF21 on brain signaling across longer testing periods and among additional populations (i.e., children/adolescents and older adults) to examine FGF21 effects on neural signaling developmentally and across the lifespan. Future studies should also investigate the effects of FGF21-linked neural signaling on food cravings and eating behavior as well as how obesity might impact these effects.

## Conclusions

In summary, our study among young adult humans reveals three important findings: 1) greater circulating FGF21 is associated with sucrose-induced dorsal striatal signaling, (2) BMI is associated with greater sucrose-induced FGF21 secretion, and (3) high vs low dietary sugar intake is associated with greater FGF21 among healthy-weight individuals. Our correlative findings support evidence in animals showing peripherally secreted FGF21 acts on the brain to regulate sugar consumption through a negative feedback loop. Heightened FGF21 responses to acute sucrose ingestion may be a compensatory mechanism to reduce the metabolic stress associated with obesity or high dietary sugar intake, and these speculations warrant further investigation.

## Supplementary Material

supinfo

## Figures and Tables

**Table 1.** Participant characteristics.

(N=68)		
Variable	Mean (SD) or N (%)	Range
BMI (kg/m^2^)	27.1 (4.9)	19.2–40.3
Age (years)	23.2 (3.8)	18.2–34.5
Sex Males Females	N=29 (43%) N=39 (57%)	
BMI Groups^a^ Healthy Weight Overweight Obesity	N=24 (35%) N=24 (35%) N=20 (29%)	
Baseline Plasma FGF21 (pg/ml)	287.8 (248.1)	5.50–1192.8
FGF21 AUC_120_ (pg/ml·min)	44153.5 (34938.8)	646.2–157519.4

a Percentages may not be equal due to rounding to nearest whole number.

## Data Availability

The datasets generated and analyzed during the current study are available from the corresponding author (K.A.P.) on reasonable request, and all brain imaging data are available in online digital repository (15).

## Study Importance

### What is already known?

- Fibroblast growth factor 21 (FGF21) has emerged as important regulator of sugar intake in humans.
- Mechanistic studies in animals demonstrate that FGF21 rises in response to ingestion of sugar and acts as a down-regulator of subsequent sweet consumption, which may be due to its effects on striatal dopamine signaling and hypothalamic neurons.

### What does this study add?

- Our study is the first to examine whether circulating FGF21 is associated with postprandial brain signaling, and the first to investigate associations between BMI and dietary sugar intake on sucrose-induced FGF21 secretion.
- We show: (1) greater circulating FGF21 is associated with sucrose-induced striatal signaling; (2) BMI is positively associated with sucrose-induced FGF21; and (3) high vs low dietary sugar intake is associated with greater FGF21 among people with healthy-weight.

### How might these results change the direction of research?

- Our correlative findings in humans support evidence in animals showing that FGF21 affects striatal signaling. Future work should investigate the effects of FGF21-linked neural signaling on food cravings and eating behavior as well as the impact of obesity on these effects.

## Abbreviations:

FGF21: fibroblast growth factor 21
BMI: body mass index
MRI: magnetic resonance imaging
ASL: arterial spin labeling
CBF: cerebral blood flow
