# Supplementary material for: FGF21 Response to Sucrose is Associated with BMI and Dorsal Striatal Signaling in Humans
Source: Obesity (Silver Spring). Author manuscript; Available in PMC 2025 Feb 5. (PMC11798647; doi:10.1002/oby.23432)
Supplement: supinfo [file NIHMS1791059-supplement-supinfo.docx]

**Supplementary Materials: FGF21 Response to Sucrose is Associated with BMI and Dorsal Striatal Signaling in Humans**

Jasmin M. Alves^1,2&^, Alexandra G. Yunker^1,2&^, Shan Luo^1,2,3^, Kay Jann^4^, Brendan Angelo^1,2^, Alexis DeFendis^1,2^, Trevor A. Pickering^5^, Alexandro Smith^1,2^, John R. Monterosso^3^, Kathleen A. Page^1,2^*

^1^ Division of Endocrinology, Department of Medicine, Keck School of Medicine, University of Southern California, Los Angeles, CA 90089.

^2^ Diabetes and Obesity Research Institute, Keck School of Medicine, University of Southern California, Los Angeles CA 90089.

^3^ Department of Psychology, University of Southern California, Los Angeles, CA 90089, USA

^4^ Mark & Mary Stevens Neuroimaging & Informatics Institute, Keck School of Medicine, University of Southern California, Los Angeles, CA 90089

^5^ Department of Preventive Medicine, Keck School of Medicine, University of Southern California, Los Angeles, CA 90089

***Correspondence:** Dr. Kathleen A. Page, MD, Associate Professor of Medicine, USC Keck School of Medicine, Division of Endocrinology, Diabetes and Obesity Research Institute, 2250 Alcazar Street; CSC 209, Los Angeles, CA 90089.

Email: [kpage@usc.edu](mailto:kpage@usc.edu)

^&^Share joint first-authorship.

**Table S1.** Plasma FGF21 levels stratified by BMI group and Added Sugar Intake

|  | Healthy Weight | Overweight | | Obesity | ^a^p-value |
| --- | --- | --- | --- | --- | --- |
| Baseline Plasma FGF21 (pg/ml) | 5.93 (0.39) | 5.85 (0.39) | | 6.81 (0.43) | 0.20 |
| Peak Sucrose induced FGF21 (pg/ml) (120min) | 7.81 (0.46) | 7.79 (0.46) | | 8.75 (0.50) | 0.29 |
| FGF21 AUC_120_ (pg/ml·min) | 32.23 (1.87) | 31.97 (1.87) | | 36.80 (2.06) | 0.16 |
|  | | | | | |
|  | **Low Added Sugar** | | **High Added Sugar** | | **^a^P-value** |
| Baseline Plasma FGF21 (pg/ml) | 5.89 (0.29) | | 6.51 (0.36) | | 0.18 |
| Peak Sucrose induced FGF21 (pg/ml) (120min) | 7.65 (0.33) | | 8.58 (0.42) | | 0.09 |
| FGF21 AUC_120_ (pg/ml·min) | 31.95 (1.38) | | 35.39 (1.72) | | 0.12 |

Data are least squares means +/- SE. ^a^ANOVA used to test for group differences in FGF21 by BMI group or added sugar intake, adjusted for sex, age, added sugar intake or BMI group respectively. Baseline plasma FGF21 (pg/ml), peak sucrose induced FGF21 (pg/ml) (120min) and FGF21 AUC_120_ (pg/ml·min) were cubic-root transformed.

**Table S2.** Correlations between Plasma FGF21 levels with Percent Calories from Added Sugar

|  | **Spearman Correlation Coefficient** | **P-value** |
| --- | --- | --- |
| **Baseline Plasma FGF21 (pg/ml)** | 0.14 | 0.27 |
| **Peak Sucrose induced FGF21 (pg/ml) (120min)** | 0.14 | 0.25 |
| **FGF21 AUC_120_ (pg/ml·min)** | 0.16 | 0.20 |

Spearman Rank Correlation Coefficient (R_s_) used because FGF21 (pg/ml), peak sucrose induced FGF21 (pg/ml) (120min) and FGF21 AUC_120_ (pg/ml·min) were not normally distributed. Correlations adjusted for age, sex and BMI group.

**Table S3.** Least squares means for FGF21 (AUC)_120_ levels following sucrose ingestion stratified by BMI group and added sugar intake. FGF21 (AUC)_120_ was cubic-root transformed. *Indicates significant difference in FGF21 response between high vs low added sugar consumption at p<0.05.

| **BMI Group** | **Added Sugar Intake** | **LS Mean (SE)** | **p-value** | **N** |
| --- | --- | --- | --- | --- |
| Healthy Weight | Low | 28.15 (2.48) | **0.04*** | 14 |
|  | High | 36.66 (2.96) |  | 10 |
|  | | | | |
| Overweight | Low | 30.25 (2.42) | 0.26 | 13 |
|  | High | 34.43 (2.63) |  | 11 |
|  | | | | |
| Obesity | Low | 36.54 (2.47) | 0.74 | 14 |
|  | High | 34.76 (4.12) |  | 6 |

**Table S4.** Correlations between Plasma FGF21 levels with Percent Calories from Added Sugar Stratified by BMI group.

|  | **Baseline Plasma FGF21 (pg/ml)** | | **Peak Sucrose induced FGF21 (pg/ml) (120min)** | | **FGF21 AUC_120_ (pg/ml·min)** | |
| --- | --- | --- | --- | --- | --- | --- |
|  | **R_s_** | **p-value** | **R_s_** | **p-value** | **R_s_** | **p-value** |
| **Healthy Weight** | 0.33 | 0.13 | 0.43 | 0.04* | 0.46 | 0.03* |
| **Overweight** | 0.01 | 0.97 | 0.13 | 0.55 | 0.15 | 0.51 |
| **Obesity** | -0.26 | 0.30 | -0.06 | 0.80 | -0.11 | 0.65 |

Spearman Rank Correlation Coefficient (R_s_) used because FGF21 (pg/ml), peak sucrose induced FGF21 (pg/ml) (120min) and FGF21 AUC_120_ (pg/ml·min) were not normally distributed. Correlations adjusted for age and sex.

*Denotes significant p-value<0.05.

**Table S5.** Associations between FGF21 and cerebral blood flow (CBF) AUC_35_ in a priori regions-of-interest (ROI) following sucrose ingestion among whole cohort in the unadjusted and fully-adjusted model (adjusted for age, sex, BMI group, added sugar group, global mean CBF (mCBF), insulin AUC_35_, and glucose AUC_35_).

| **Brain ROI** | **Model** | **β Estimate (95% CI)** | **p-value** |
| --- | --- | --- | --- |
| **Dorsal Striatum** | Unadjusted | -9.12 (-16.46, -1.78) | **0.02*** |
|  | Fully-adjusted | -7.52 (-14.38, -0.65) | **0.04*** |
| **Hippocampus** | Unadjusted | -7.14 (-17.39, 3.10) | 0.18 |
|  | Fully-adjusted | -5.42 (-14.21, 3.37) | 0.23 |
| **Hypothalamus** | Unadjusted | -2.09 (-10.92, 6.74) | 0.64 |
|  | Fully-adjusted | 0.33 (-8.26, 8.91) | 0.94 |
| **Insula** | Unadjusted | -12.60 (-24.48, -0.72) | **0.04*** |
|  | Fully-adjusted | -8.23 (-18.64, 2.19) | 0.12 |
| **Nucleus Accumbens** | Unadjusted | -10.98 (-22.31, 0.35) | 0.06 |
|  | Fully-adjusted | -9.17 (-20.92, 2.57) | 0.13 |

**Table S6.** Associations between FGF21 and cerebral blood flow (CBF) AUC_35_ in a priori regions-of-interest (ROI) following sucrose ingestion, stratified by BMI status in the unadjusted and fully-adjusted model (adjusted for age, sex, BMI group, added sugar group, global mean CBF (mCBF), insulin AUC_35_, and glucose AUC_35_).

| **Brain ROI** | **BMI Status** | **Model** | **β Estimate (95% CI)** | **p-value** |
| --- | --- | --- | --- | --- |
| **Dorsal Striatum** | Healthy Weight | Unadjusted | -16.41 (-29.27, -3.55) | **0.02*** |
|  |  | Fully-adjusted | -17.06 (-27.97, -6.15) | **0.007*** |
|  | Overweight | Unadjusted | -5.92 (-17.19, 5.35) | 0.31 |
|  |  | Fully-adjusted | -3.73 (-19.71, 12.25) | 0.65 |
|  | Obesity | Unadjusted | -4.22 (-19.52, 11.08) | 0.60 |
|  |  | Fully-adjusted | -14.4 (-29.78, 0.98) | 0.09 |
| **Hippocampus** | Healthy Weight | Unadjusted | -22.94 (-38.94, -6.93) | **0.01*** |
|  |  | Fully-adjusted | -25.21 (-41.08, -9.33) | **0.007*** |
|  | Overweight | Unadjusted | -12.20 (-29.20, 4.80) | 0.17 |
|  |  | Fully-adjusted | -18.13 (-37.62, 1.36) | 0.09 |
|  | Obesity | Unadjusted | 14.35 (-4.73, 33.42) | 0.16 |
|  |  | Fully-adjusted | 1.22 (-15.62, 18.06) | 0.89 |
| **Hypothalamus** | Healthy Weight | Unadjusted | -2.21 (-16.15, 11.73) | 0.76 |
|  |  | Fully-adjusted | -7.86 (-22.92, 7.20) | 0.32 |
|  | Overweight | Unadjusted | -1.60 (-17.83, 14.63) | 0.85 |
|  |  | Fully-adjusted | -11.53 (-34.87, 11.82) | 0.35 |
|  | Obesity | Unadjusted | -4.18 (-23.13, 14.76) | 0.67 |
|  |  | Fully-adjusted | -9.21 (-26.14, 7.72) | 0.31 |
| **Insula** | Healthy Weight | Unadjusted | -22.60 (-42.45, -2.74) | **0.04*** |
|  |  | Fully-adjusted | -18.37 (-35.53, -1.22) | **0.05*** |
|  | Overweight | Unadjusted | -10.70 (-29.34, 7.94) | 0.27 |
|  |  | Fully-adjusted | -7.57 (-36.02, 20.87) | 0.61 |
|  | Obesity | Unadjusted | 0.21 (-25.80, 26.21) | 0.99 |
|  |  | Fully-adjusted | -7.74 (-31.40, 15.93) | 0.53 |
| **Nucleus Accumbens** | Healthy Weight | Unadjusted | -13.22 (-31.64, 5.19) | 0.17 |
|  |  | Fully-adjusted | -17.45 (-38.74, 3.83) | 0.13 |
|  | Overweight | Unadjusted | -13.29 (-29.77, 3.19) | 0.13 |
|  |  | Fully-adjusted | -18.87 (-42.46, 4.72) | 0.14 |
|  | Obesity | Unadjusted | -14.29 (-40.37, 11.78) | 0.30 |
|  |  | Fully-adjusted | -12.74 (-45.82, 20.34) | 0.46 |

**Figure S1. Histogram of Percent Calories from Added Sugar**

**
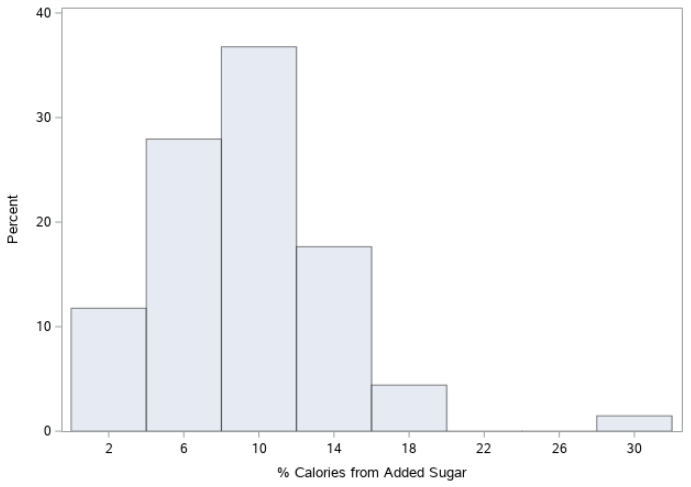
**

Bins of percentages of participants who consumed percent calories from added sugar.

**Figure S2.** **Relationship between high vs low added sugar intake and sucrose-induced plasma FGF21 response**

High compared to low added sugar intake was associated with a greater FGF21 response to acute sucrose ingestion among people with healthy weight, but results were attenuated in participants with overweight and obesity. Data are expressed as raw/unadjusted mean ± SEM for visual purposes, but all statistical analyses were based on cubic root transformed FGF21 values and adjusted for covariates. *Indicates significant difference in FGF21 response between high vs low added sugar consumption at p<0.05.
